# A computer-based incentivized food basket choice tool: Presentation and evaluation

**DOI:** 10.1371/journal.pone.0210061

**Published:** 2019-01-10

**Authors:** Jonathan Spiteri, Jonathan James, Michèle Belot

**Affiliations:** 1 Faculty of Economics, Management and Accountancy, University of Malta, Msida, Malta; 2 Department of Economics, University of Bath, Bath, United Kingdom; 3 Department of Economics, European University Institute, Florence, Italy; University of Sheffield, UNITED KINGDOM

## Abstract

**Objective:**

To develop and evaluate a low-cost computer-based tool to elicit dietary choices in an incentive compatible manner, which can be used on-line or as part of a laboratory study.

**Methods:**

The study was conducted with around 255 adults. Respondents were asked to allocate a fixed monetary budget across a choice of around a hundred grocery items with the prospect of receiving these items with some probability delivered to their home by a real supermarket. The tool covers a broad range of food items, allows inference of macro-nutrients and calories, and allows the researcher to fix the choice set participants can choose from. We compare the information derived from our incentivized tool, and compare it to alternative low-cost ways of measuring dietary intake, namely the food frequency questionnaire and a one-shot version of the 24-hour dietary recall, which are both based on self-reports. We compare the calorie intake indicators derived from each tool with a number of biometric measures for each subject, namely weight, body-mass-index (BMI) and waist size.

**Results:**

The results show that the dietary information collected is only weakly correlated across the three methods. We find that only the calorie intake measure from our incentivized tool is positively and significantly related to each of the biometric indicators. Specifically, a 10% increase in calorie intake is associated with a 1.5% increase in BMI. By contrast, we find no significant correlations for either of the two measures based on self-reports.

**Conclusion:**

The computer-based tool is a promising new, low-cost measure of dietary choices, particularly in one-shot situations where such behaviours are only observed once, whereas other tools like 24-hour dietary recalls and food frequency questionnaires may be more suited when they are administered repeatedly. The tool may be useful for research conducted with limited time and budget.

## Introduction

Academic interest in nutrition has increased dramatically in recent years and across a variety of disciplines spanning Social and Medical sciences. One of the major challenges encountered by researchers and practitioners across all disciplines is related to the measurement of dietary intake among people. This challenge is important to tackle because a proper measurement of dietary intake is crucial to the design and evaluation of policy interventions. There exist a variety of tools that have been evaluated and validated, going from simple surveys to more sophisticated methods like the 24-hour dietary recall [1], a questionnaire-based tool whereby subjects are asked to provide details regarding all food and drink items consumed within the last 24 hours, including portion size, brand, side-dishes, sauces, snacks and condiments. These recalls can be either administered by qualified nutritionists or self-administered, and are considered to be the gold standard for nutrition-based studies. The major benefit from such methods is that they target actual dietary intake. However, the reliability of this tool depends on memory accuracy and truthful reports. It also sometimes requires having multiple entries for each person over several days, in order to obtain a truer picture of the subject’s actual diet, thereby making such measures somewhat costly. In addition, the problems associated with self-reported data are particularly acute for dietary recalls since research has shown that people who are either obese or at risk of obesity are more likely to under-report their true dietary intake, which may limit the reliability of any results obtained [2].

On the other side of the spectrum, we find methods that are based on purchases rather than actual intake, such as those using scanner data (such as [3, 4] and [5]). The difficulty here is that there might be a discrepancy between purchase and consumption behavior, and it is more difficult to separate individual consumption from household consumption.

This paper contributes towards this methodological debate by proposing a new incentivized method of information elicitation which can be administered relatively inexpensively either in a laboratory or online setting. In essence, the idea behind our proposed approach is to incentivize respondents to provide accurate responses, building on the substantial existing literature on real choice experiments across several fields, including nutrition (e.g. [6]; [7]). Our approach fits in spirit with the approach of recent studies that experimentally evaluate the effects of interventions on dietary choices by looking at a limited set of choices like lunch or snack choices (e.g. [8]; [9]). These choices are obviously not a reflection of the entire spectrum of dietary choices across an entire day. Indeed, it may be the case that any healthy choices or calorie reductions recorded in terms of meal choices may be compensated by increased consumption of unhealthy or higher-calorific options in another meal, as partially observed in [10]. The idea here is to collect information about food purchases in a low-cost and efficient way. The tool resembles similar tools that have been developed in other contexts, such as [11] and [12] who use a online shopping tool including a wide range of products (including non food items) to study environmentally friendly purchases.

The tool we proposed is inspired by current and common online supermarket interfaces. As part of this tool, subjects are allocated a fixed budget and are asked to select among a range of around a hundred food and drink items typically found at a local supermarket such as fruit and vegetables or ready meals. The range has been selected from real on-line supermarket items and includes popular items. Participants are incentivized to make ‘honest’ choices by having a chance of receiving their basket at home. All participants were provided with a participant information sheet and consent form where we state that ‘You will also get a chance to receive a £30 basket of food items to be delivered to you.’ Therefore participants were fully aware that they had this chance of winning the food items. All choices are recorded, with the tool also containing individualized nutritional data for each food and drink item available from the online supermarket, including calorie content, fats and sugar, thus allowing the researcher to calculate the nutritional composition of each subject’s choices.

One important feature of the tool is that it allows researchers to determine the food choice set. That is, researchers can determine what foods people can choose from. This is potentially useful because there is a concern that dietary choices may be limited by supply—the idea that food deserts exist and make it hard for certain groups of the population to access certain foods, such as fresh fruit and vegetables. With this tool, one can fix the choice set and study the demand conditional on this choice set.

To evaluate the usability and functionality of our proposed food choice tool, we invited low income individuals (n = 255) to our laboratory. Our focus on low income individuals stems from the well documented socio-economic gradient in chronic diseases ([13]). We are interested in comparing the information obtained with alternative ways one could collect information with limited time constraints and budgets. We also collected information using two alternative measures of dietary intake, namely a Food Frequency Questionnaire (FFQ), a one-shot version of a self-administered 24-hour dietary recall. We first compare the nutritional profiles of each subject across the three tools to assess whether the results obtained are similar to one another. Secondly, we correlate the total calorie intake derived from each of the three dietary measures with a number of subject-specific biometric measures, weight, body-mass index (BMI) and waist size, all of which have a well-documented relationship with dietary intake [14].

Overall, we find that the measures are not strongly correlated. We find no significant relationship between the calorie, fat and saturated fat intakes inferred from the three methods. When comparing the nutrient content inferred from each tool with the biometric measures, the only statistically-significant and positive results obtained are for the calorific intake measures obtained via our food choice tool. By contrast, we do not find any statistically-significant relationship between calorie intake measures from the FFQ or 24-hour dietary recall and our various biometric measurements. This lack of correlation may be due to a systematic bias in the reported levels of calorific intake by participants (similar to that suggested in [15]) and a predominance of noisy observations which would raise standard errors and thus suppress significance levels.

Our incentivized food choice tool has a number of important advantages. First, it provides a quick and relatively inexpensive way of capturing people’s dietary choices, without having to repeatedly administer a survey in order to obtain multiple data points. This is particularly important in field work where multiple surveys may be both infeasible and prohibitively costly. Second, the online supermarket-based interface allows for a much broader array of food and drink items to be selected, encompassing all main meals and snacks for up to an entire week (depending on the budget allocated), thereby providing a more complete and representative record of the participants’ typical purchasing behavior. Third, by incentivizing participant responses by delivering the food and drink choices for all or a selection of participants, the tool can help to circumvent any issues associated with inaccuracies and under-reporting prevalent in self-reported survey measures [16]. Fourth and as already mentioned, it allows researchers to fix the choice set.

## Related literature

This paper contributes to the growing literature on the use and development of various measures of dietary choices. The nutrition and epidemiology literature has proposed several tools which could be utilized in order to gauge people’s diets. The 24-hour dietary recall is perhaps the most widely-used method of eliciting intake patterns by nutritionists and epidemiologists [17], particularly since people’s memory of what they actually consumed may be more precise, although as pointed out by [18] the variability of intake from day to day may hamper the representativeness of one-day recalls. In fact, research by [19] suggests that averaging people’s energy intake over a 3-day period would provide the the highest level of accuracy from 24-hour dietary recall data.

Another popular method for eliciting dietary patterns is the Food Frequency Questionnaire (FFQ), which typically asks respondents to indicate the frequency with which they have consumed a fixed list of food and drink items over the past month [20]. The benefits of using the FFQ are largely based around its practicality as well as its ability to minimize day-to-day variation in diet since it is nominally a one-shot measure [21], although doubts regarding its accuracy have been widely-cited in the literature due to the closed-nature of the questions asked and the possibility of errors or under-reporting [22].

Various studies have been conducted in order to assess the validity of both the 24-hour dietary recall and the FFQ. When it comes to capturing food intake, the results are somewhat mixed, since although some find no significant differences between the two (e.g. [23]), others report that the 24-hour dietary recall is more accurate, particularly among cohorts with a high obesity prevalence (e.g. [24]). In terms of their correlation with anthropometric measures, again we find decidedly conflicting results for both the dietary recall and the FFQ, since in either case some studies find a positive relationship between reported calorie intake and body measurements (e.g. [25]; [26]), whereas in other cases no correlation is reported (e.g. [27]; [28]). Several other methods have also been proposed, like dietary food logs and diet history, each with their own pros and cons, although the pervasiveness of the aforementioned two methods remains [29].

By contrast, our proposed food choice tool seeks to elicit dietary choices by incentivizing them. Previous work in the literature has also sought to utilize revealed preferences in order to derive information regarding people’s dietary choices. For example, [3], [4] and [5] all make use of highly-detailed panel data based on consumers’ scanned supermarket purchases in order to obtain estimates of household dietary choices across a variety of nutrients and food categories, while controlling for prices. In addition, several experiments (both in the lab and in the field) have relied on incentivized or real-world choices ([30]; [31]; [32]).

In particular, our paper is closely-related to the branch of the sizeable literature of *real choice experiments* (RCEs) in the context of dietary choices ([33]; [34]; [35]). For example, [36] use a randomized choice experiment in order to assess participants’ willingness to pay for five different types of quarter-pounder steaks of varying quality and characteristics, while [37] used similar methods in order to elicit preferences regarding the color of salmon. Our paper builds on these ideas by developing a unified, intuitive tool to assess dietary choices, where the setting is familiar and choices are realizable, thereby incentivizing true responses. This tool can be utilized in a variety of settings, from lab and field experiments to online administration, and represents a relatively inexpensive way of eliciting choices when compared to other tools like the 24 hour dietary recall.

Our paper also contributes to the well-established literature on survey design and information elicitation techniques. The fundamental driving force behind the emergence of this literature is the skepticism surrounding self-reported responses (e.g. [38]). In fact, various authors like [16] have pointed out the significant potential drawbacks of relying on self-reported survey methods for data elicitation, including respondent inability to recall vital information, possible dissonance between respondent actions or beliefs and his/her self-image, leading to biased responses, and problems with understanding the questions asked.

This issue is particularly relevant since, as pointed out in [39], response errors or omissions are typically correlated with key individual characteristics and behavioral tendencies, thus potentially introducing biases in any inferred results. Within this context, several alternative elicitation techniques for surveys have been proposed across several disciplines, including open-ended (as opposed to closed) questions and bidding games [40], randomized response techniques [41] and item count techniques [42]. The concept of our tool is based on revealed preferences, building on the theoretical underpinnings provided in [43] regarding the ability of material incentives to generate economically-consistent and truthful behavior.

## The food choice tool

The incentivized information-elicitation method developed in this paper is a computer-based food choice tool. The tool is open source and available to all researchers. A copy, including full user instructions, can be dowloaded from the following website: https://sites.google.com/site/jonathanspiteriphd/food-choice-tool. As discussed earlier, the aim of this tool is to elicit reliable and accurate dietary patterns from respondents, which in turn can be used to evaluate the effectiveness or otherwise of various interventions aimed at changing these behaviors. This tool has a simple user interface that is based on an online supermarket with a wide array of food and drink items that can be selected. The items are organized under six main headings (each represented by a separate tab at the bottom of the page): fruit and vegetables, meat and fish, bread and grains, confectionery and snacks, ready meals and drinks. The food and drinks have been selected as the most popular items in their respective categories as of March 2016 (the month of the tool’s inception), as listed on one of the UK’s leading supermarkets’ official website. In total our food choice tool contains 120 different items; these are listed below in Table 1, while Table 2 provides the average nutrient content (per 100g) of each of the six food categories.

**Table 1:** List of food and drink items in the food choice tool.

**Fruit and Veg**	**Meat and Fish**	**Bread and Grains**
Royal Gala Apples, x5	Chicken Breast Fillets, 460g	Sliced White Bread, 800g
Fairtrade Bananas, x5	Chicken Kiev, Garlic, x2	Sliced 50/50 Bread, 800g
Oranges, x6	Chicken Goujons, 245g	Sliced Wholemeal Bread, 800g
Red Grapes, 500g	Chicken Pie, 550g	White Baguettes, x2
Conference Pears, x4	Beef Rump Steak, 250g	Brown Baguettes, x2
Raspberries, 150g	Beef Mince, 500g	White Rolls, x8
Strawberries, 400g	Steak Burgers, 340g	Wholemeal Rolls, x8
Peaches, x4	Steak and Ale Pie, 550g	Crumpets, 400g
Kiwi Fruit, x4	Pork Chops, 450g	Plain Naan Bread, 260g
Lemons, x5	Pork Sausages, 400g	Brown Soda Bread, 400g
Cherry Tomatoes, 650g	Mini Pork Pies, 300g	Tortilla Wraps, x8
White Mushrooms, 300g	Smoked Bacon, 300g	Basmati Rice, 1kg
Maris Piper Potatoes, 2.5kg	Lamb Chops, 275g	Brown Basmati Rice, 1kg
Sweet Potatoes, 1.25kg	Salmon fillets, 240g	Penne Pasta, 1kg
Mixed Peppers, x3	Cod fillets, 250g	Wholewheat Penne, 1kg
Carrots, 1kg	Sea Bass Fillets, 180g	Spaghetti, 500g
Onions, 1kg	King Prawns, 150g	Wholewheat Spaghetti, 500g
Fine Beans, 200g	Breaded Cod, 350g	Cous Cous, 1kg
Broccoli, 335g	Smoked Haddock with Cheese, 400g	Quinoa, 300g
Sweetcorn, x2	Salmon en Croute, 380g	Egg Noodles, 375g
	Cod Fish Fingers, 480g	
	Mackerel in Garlic Butter, 340g	
**Confectionery**	**Ready Meals**	**Drinks**
Dairy Milk Chocolate, 200g	Cheese & Tomato Pizza, 10”	Blackcurrant Squash, 850ml
70% Dark Chocolate, 100g	Pepperoni Pizza, 10”	Orange & Passion Fruit Drink, 4x275ml
Mars Bars, x4	Beef Lasagne, 430g	Coconut Water, 1L
Snickers, x4	Macaroni Cheese, 430g	Sports Drink, 1L
M&M’s Peanut, 165g	Chicken & Bacon Pasta Bake, 430g	Energy Drink, 1L
Nutella, 400g	Cottage Pie, 450g	Ginger Beer, 1.5L
Starmix Candy, 215g	Beef Stew, 450g	Tonic Water, 1L
Jelly Babies, 190g	Chicken Tikka & Rice, 500g	Coke, 1.75L
Marshmallows, 200g	Beef Burrito, 400g	Irn Bru, 2L
Chocolate Chip Brioche, x8	Chicken Chow Mein, 450g	Fanta, 2L
Chocolate Digestives, 300g	Beef Satay, 380g	Orange Juice, 1L
Chocolate HobNobs, 262g	Chicken Ramen, 380g	Mango & Passion Fruit Smoothie, 750ml
Shortbread Fingers, 400g	Pigs in Blankets, 260g	Still Water, 6x500ml
Custard Creams, 400g	Vegetarian Cannelloni, 430g	Chocolate Milkshake, 1L
Chocolate Cookies, 175g	Vegetable Spring Rolls, 60g	Soya Milk, 1L
Pringles Original, 190g	Vegetable Biryani, 500g	
Salt & Vinegar Chips, 150g	Tomato & Mozzarella Bake, 430g	
Cheese & Onion Crisps, 6x25g	Lentil Cottage Pie, 400g	
Corn Chips, 200g	Mushroom Risotto, 430g	
Croissants, x8	Tomato Soup, 600g	

*Note*: In this table we list all of the 120 food and drink items included in our proposed Food Choice tool, under each respective category.

**Table 2:** Average nutrient content per Category (per 100g).

	Calories	Fat	Sat Fat	Carbs	Sugar	Protein
Fruit and Veg	47.4	0.5	0.1	9.7	7.2	1.4
Meat and Fish	233.2	13.9	4.9	7.5	0.8	19.4
Bread and Grains	212.1	1.9	0.4	40.6	2.4	7.0
Confectionery	471.9	22.5	9.1	59.4	33.1	6.4
Ready Meals	157.7	6.9	2.6	15.3	2.6	7.1
Drinks	40.5	0.2	0.1	9.9	9.2	0.6

*Note*: This table summarizes the average nutrient content, per 100g, of each of the six food and drink categories used as part of our proposed food choice tool. We consider six key nutrients, namely calories (in kcal), fat, saturated fat, carbohydrates, sugar and protein.

**Fig 1:**
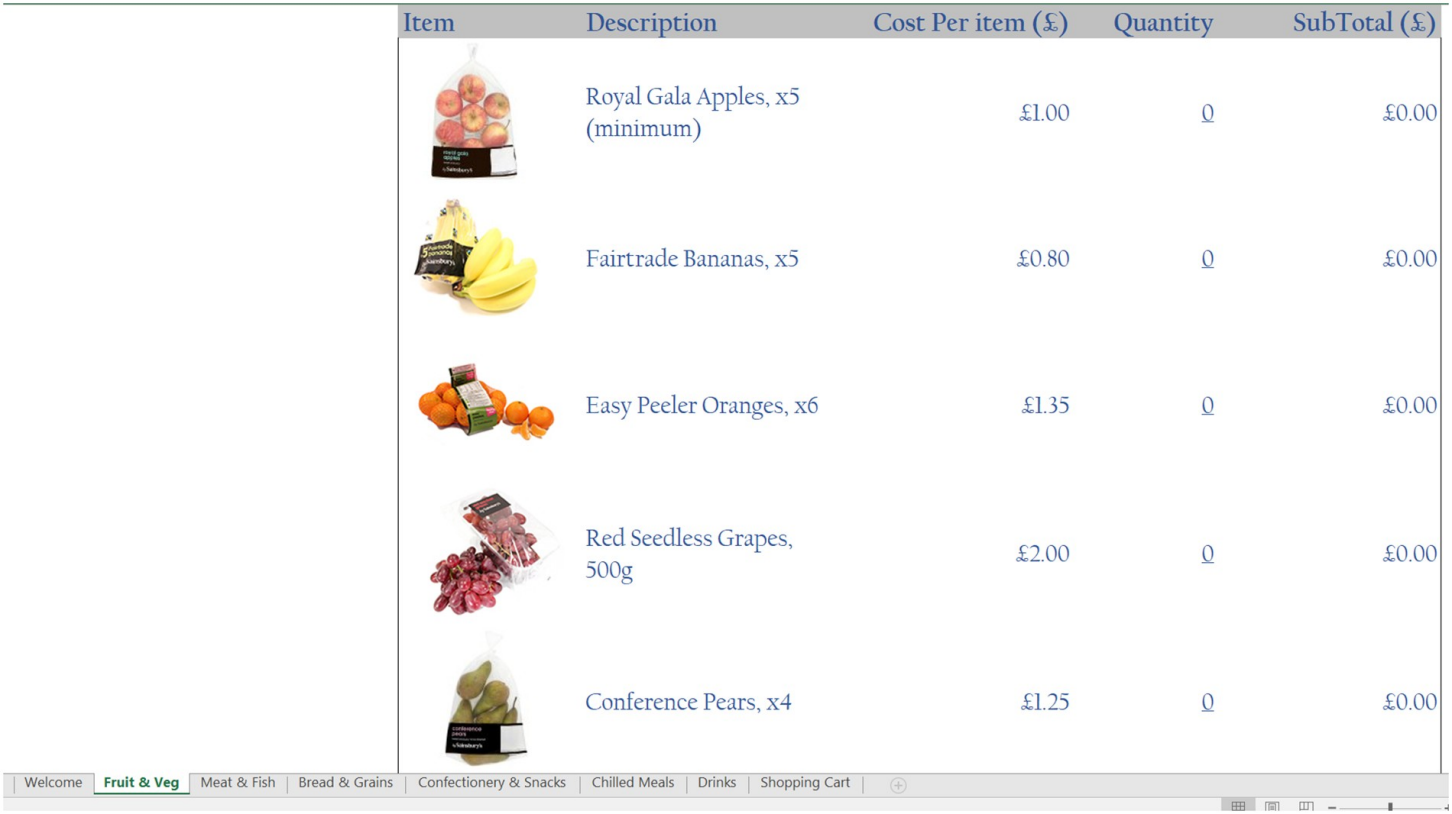
Screenshot 1 of the food choice tool.

**Fig 2:**
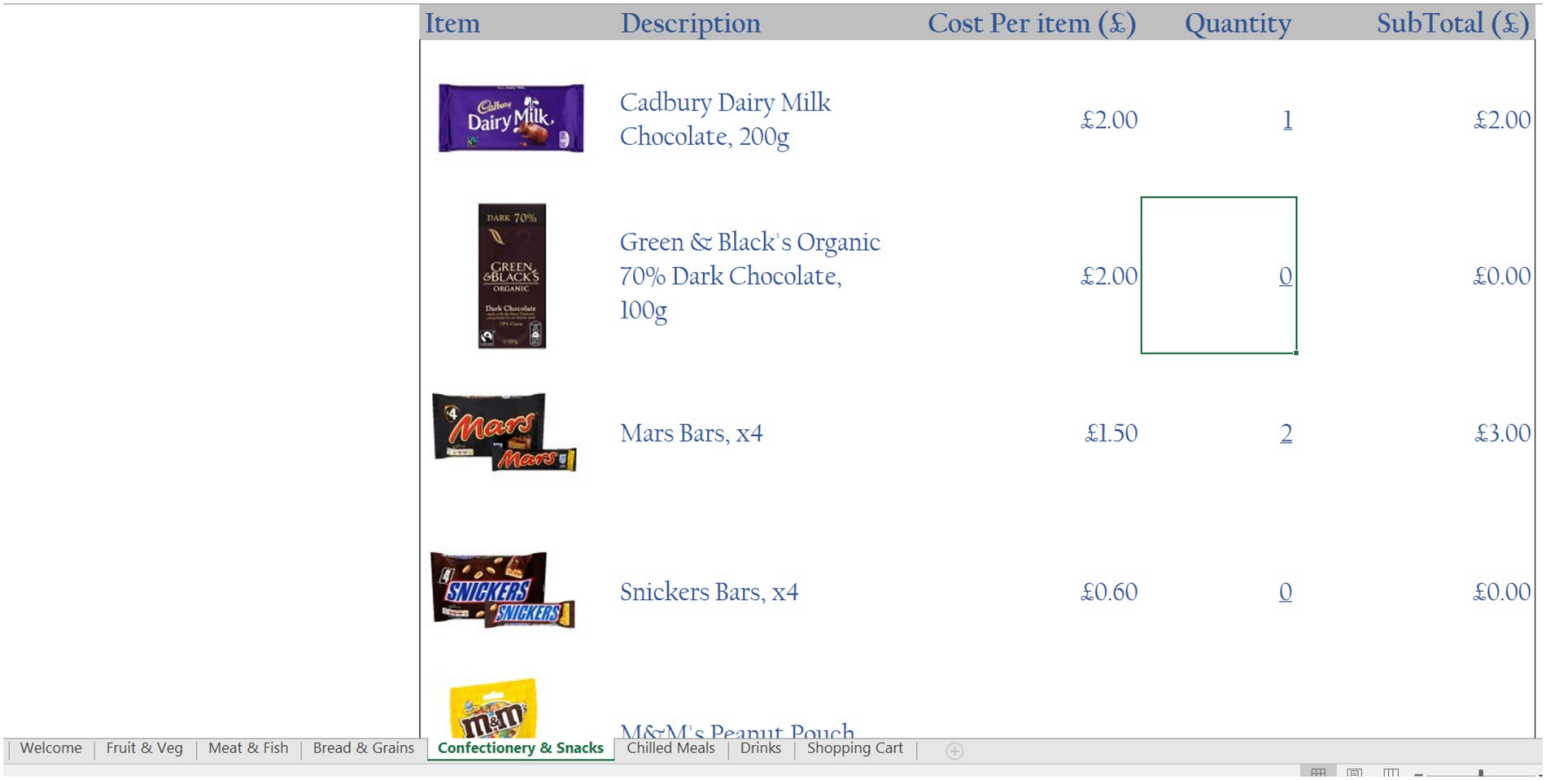
Screenshot 2 of the food choice tool.

**Fig 3:**
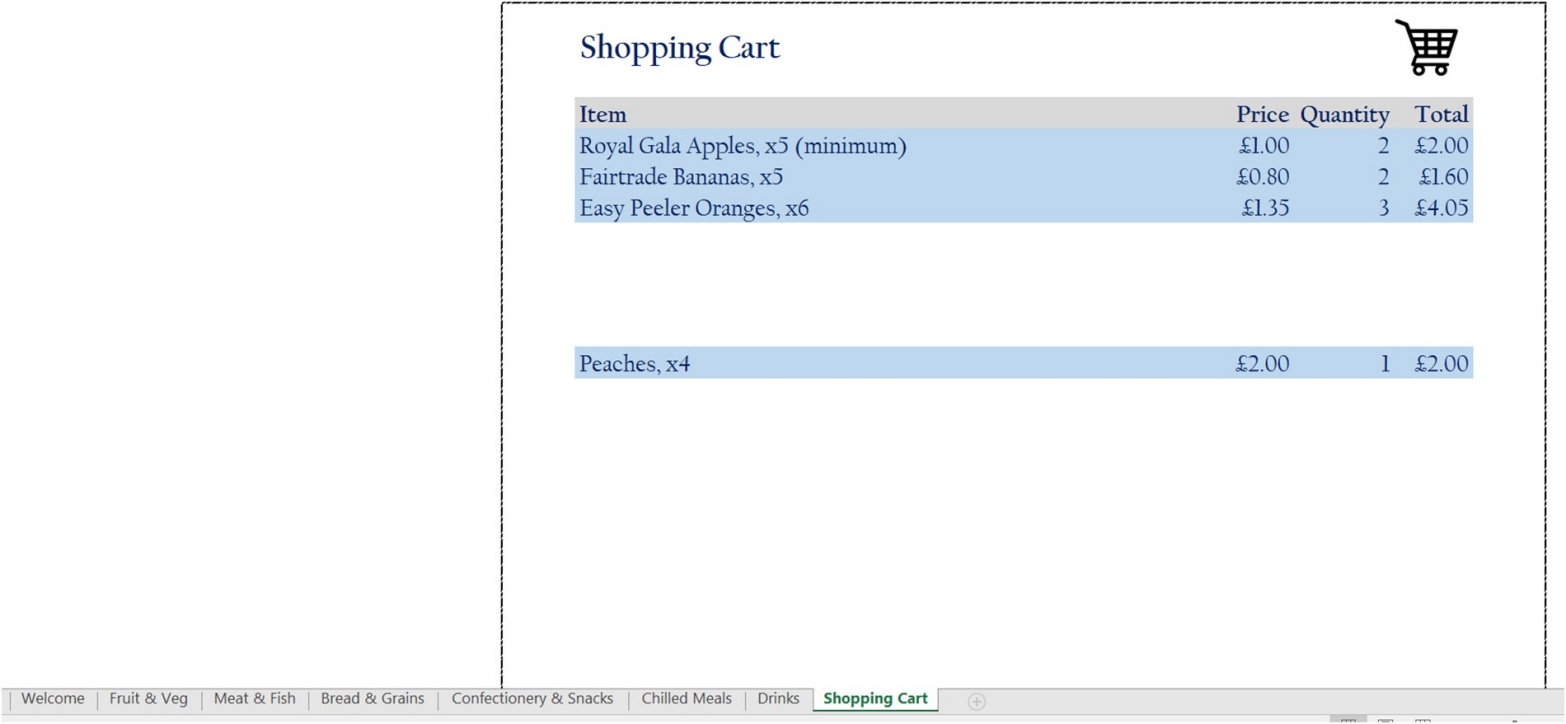
Screenshot 3 of the food choice tool.

**Fig 4:**
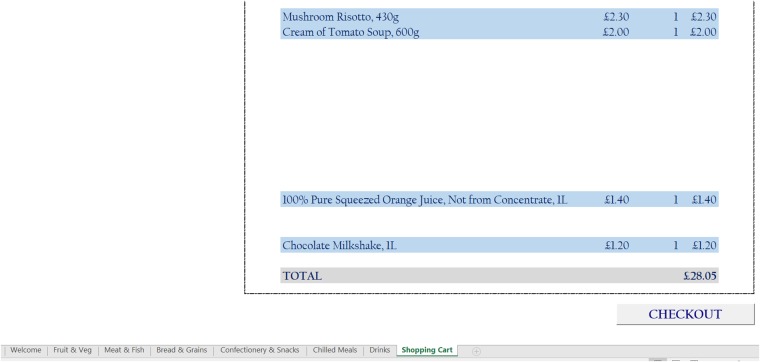
Screenshot 4 of the food choice tool.

In the food choice tool, each item is listed along with a thumbnail picture and its price, which is the actual price as at March 2016 based on listings on the same leading supermarket’s website, thereby ensuring that the prices reflect actual high street prices. The tool operates like a basic online supermarket, where respondents can select any food or drink item by specifying a quantity in the space provided next to each item. There are no restrictions on food choices, neither in terms of which items can be selected, nor in terms of the quantity of each item that can be picked. A number of screenshots of the tool, showing the various food and drink items on offer as well as the user interface, are shown in Figs 1, 2, 3 and 4.

The last tab/category shown in the tool is the ‘Shopping Cart’ page, which lists all of the items (and quantities) selected by the user, together with the total amount ‘spent’. It is relatively straightforward to set a fixed budget that participants can spend; this should ideally reflect average household weekly or periodic spending at the supermarket in order to garner a more complete picture of participants’ food intake. The system will then notify participants whenever this budget has been exceeded via a pop-up dialog box, inviting them to delete any items as appropriate. Once the participant is satisfied with the choices made, he/she can press a button at the bottom of the ‘Shopping Cart’ tab labeled ‘Checkout’ in order to save the choices and close the program. Note that if the pre-set budget has been exceeded, the system will not close down and instead notify the user to modify his/her selection.

Incentivizing the choices made is one of the main features of this tool, in order to avoid the pitfalls associated with self-reported or survey based information elicitation methods. The appeal of our food choice tool lies in the fact that incentives can be flexible and thus tailored to suit a variety of research budgets and logistical realities. The most straightforward and direct method would be to inform all users beforehand that their choices would be ordered from an actual supermarket and delivered to them within a few days, depending on their requirements. However, this may prove to be infeasible, both financially and logistically, as it requires ordering each basket and organizing food deliveries for each participant. Therefore, we propose an alternative solution whereby once all choices have been made, one or several participants are picked at random, with only their chosen baskets ordered and delivered. This randomized scheme mitigates against the problem of financial and logistical infeasibility. Moreover the literature on random lottery incentive systems in experimental economics shows that the choices made under this regime are not systematically different from those made under a full-pay system ([44]; [45]).

Given getting the basket is a lottery. Those in groups with fewer participants would have had a greater probability of receiving their basket, conversely those in an experimental session have a smaller chance and may pay less attention to their choices or may felt there was a good chance of not paying the consequences of mis-reporting their purchases. We find a very small and statistically insignificant relationship between group size and the number of calories in their basket suggesting the uncertainty surrounding winning the basket was not an influential factor.

The food choice tool records all food and drink choices made by each user, both in terms of quantities and amounts spent per item/category. Crucially, the tool also contains detailed nutritional data for each of the 120 items listed, including information regarding total calorie content, total fats, saturated fats, carbohydrates, sugar, salt, protein and fibre, both as aggregates and also per 100g. Therefore, our proposed food choice tool can also measure the nutritional composition of each user’s food choices, thereby providing a record of their individual dietary patterns. This tool provides clear benefits over other self-reported measures by incentivizing people’s responses to ensure greater reliability of data, while also improving the representativeness of food choices due to its supermarket interface and budgetary allowance, meaning that selected baskets would reflect multiple meals as opposed to one-shot meals or snacks.

Although our proposed tool has various important benefits, there are important specific aspects, which may turn into shortcomings depending on the question one is interested in. First, our tool focuses on *planned* food expenditures rather than impulse purchases that yield immediate gratification, given that the winning basket would not be immediately delivered. Participants were explicitly instructed to shop as they normally would for their weekly shopping. In order to mitigate the influence of what participants had in stock at home on their choices using the tool the basket chosen was delivered two weeks after the experimental session—participants were informed about that when making their choices. A significant body of work has shown how these two types of food expenditure can differ ([46]; [47]), and in particular how impulse purchases are associated with self-control failures and thus spending on unhealthy items ([48]; [49]). Thus, it may be the case that the estimates of dietary intake obtained via our tool may under-represent the actual amount of unhealthy food consumed by respondents. However, [50] find little difference between purchases in the lab and shopping outside of the lab.

Second, this is not necessarily a normal shopping experience given the limited supply of items and unknown store brand. However, the tool is flexible such that more items can be added if the researcher wishes. Having 120 items is limited compared to the regular shopping experience, this may result in the tool not being application or very good at capturing certain populations with specific diet requirements such as vegetarians or vegans. The limited number of items may also result in a baskets chosen that have smaller variance relative to a shopping tool with a wider range of products.

Third, it is not clear that this provides an accurate measure of consumption. This is also the case, however, for studies that use scanner data. Finally, the tool does not necessarily capture individual behavior, in contrast to the food frequency questionnaire and the 24-dieary recall where the participants are asked about their own behavior. We cannot enforce that individuals only purchase items for their own consumption. While this is a limitation, researchers can explicitly ask individuals to only shop for themselves. That the supermarket basket possibly reflects the preferences of other people in the household is relevant, but should weaken the correlations between the items purchased and the anthropometric measurements.

In the version of the tool we propose here, participants do not have access to the nutritional information for any of the food and drink items; rather, the food choice tool only displayed a thumbnail image of each item, together with a short description and its price (in £). This design differs somewhat from the standard grocery shopping experience, both in-store and online, since typically consumers would have access to each product’s nutritional information, and would be able to consult this information prior to making their purchasing decisions. We opted to omit such information in order to expedite the food selection process in the lab, thereby enabling a relatively quick collection of information. Furthermore, evidence suggests that people do not regularly read nutritional labels when purchasing their food and drink items from supermarkets, particularly when it comes to familiar and/or repeat purchases (e.g. [51]; [52]). This said, it would be relatively simple to include nutritional or other relevant information in the tool if one would wish to do so.

## Experimental design

Having laid out the key aspects of our proposed tool, we now proceed to compare it to other leading tools used by nutritionists and epidemiologists. To this end, we ran a laboratory-based experiment whereby participants were asked to complete a food frequency questionnaire (FFQ), a 24-hour dietary recall, as well as our supermarket-based food choice tool. We then compare the measures of nutritional intake derived from each tool, and relate them to a number of biometric indicators namely body-mass index (BMI), weight and waist size. The Ethics Committee of the University of Edinburgh approved this study.

The experiment was part of a wider study on health and nutrition, which was held at the Behavioural Laboratory at the University of Edinburgh (BLUE). Briefly, the initial wave of the study, which ran in June 2016, focused on the impact of different types of health information, as well as time availability, on food choices. As described in a different paper ([53]) we sought to compare the impact on dietary choices resulting from the provision of no health information (our control group), the provision of generic health information on heart disease and diabetes, derived from the UK’s National Health Service (NHS) and Harvard Medical School, as well as the provision of tailored health information group, who were asked to undertake a computer-based health assessment that provided personalized information regarding their individual risk of contracting heart disease and diabetes relative to the average person their age and gender living in Scotland. Note that in this study, the measure of dietary choices used was derived from our incentivized food choice tool, which was completed following the health information stage. In addition, we also looked at how varying the time available for participants to make their food and drink choices from our food choice tool influenced the nutritional composition of their choices.

The second wave of the study, which is relevant to this paper, ran from Monday 12^th^ to Friday 16^th^ September 2016, and was also held at the BLUE lab. Each day consisted of four time slots: 9.30am-11am, 11.30am—1pm, 2.30pm—4pm, 5.30pm—7pm, and participants were able to indicate their preferred time slot(s) beforehand. We conducted the experiment in 20 identical sessions of up to 18 individuals per session, spread over 5 consecutive days. Participants had received an information leaflet in advance at their home address as part of the wider study, which kicked-off in June 2016, and which also contained some information regarding this phase of the study, as well as a consent form. All procedures were done in accordance with the ethical guidelines established by BLUE and the University of Edinburgh’s School of Economics, and the study was granted full ethical approval beforehand.

### Sample and recruitment procedure

The sample for our experiment consisted of 255 participants from low-income backgrounds (< £26,500 annual income) living in the surrounding precincts of the University of Edinburgh’s main campus. More specifically, participants had to satisfy the following eligibility criteria:

- Must be over 18 years of age;
- Must live in Edinburgh;
- Must be fluent in English;
- Must have an annual household income below £26,500;
- Must not be currently undertaking any regular medical treatment;
- Must not be pregnant.

We recruited participants using various advertisement channels, such as information leaflets delivered by post to home addresses in the more deprived neighborhoods in the vicinity of the University, online advertisements and promotional emails. All participants were given £50 compensation for participating in this study.

### Procedure

Upon arriving at the BLUE lab, all participants were asked to measure their height and weight using the equipment provided (assistance was provided where necessary), and were also provided with individual tape measures in order to record their waist size. All participants were provided detailed instructions on how to measure their waist with specially designed tape measures—they were told that the tape should be placed around their abdomen, in line with their belly button, and a reading taken when the tape is snug but does not compress the skin. Assistance by members of the laboratory team was provided whenever necessary. Height measurements were taken using a stadiometer, which was operated by a member of our team. Weight was recorded using medical-grade scales, operated again by team members within the lab. In all instances, readings were taken by the participants themselves in order to protect the privacy and anonymity of the participants, in line with the ethical requirements of the study (they wrote down the numbers), although once again assistance was provided whenever individuals struggled with their readings. We appreciate that this strategy may lead to potential measurement errors in the anthropometrics. Nonetheless, various authors have shown that, despite the potential for errors, self-reported measurements are still valid and reliable estimates of people’s body measurements, and particularly if used as correlates with obesity-related behaviours and choices (e.g. [54]; [55]; [56]). They were then directed to an individual computer in the laboratory. Participants were then asked to fill out an initial computer-based questionnaire which included questions related to demographics, socio-economic background, education, employment status, as well as various questions related to their prior knowledge regarding health, nutrition and their own health status. A snapshot of our sample’s main characteristics is provided below in Table 3.

**Table 3:** Baseline characteristics of sample.

	Mean	Std. Dev.
Male	0.38	0.49
White	0.88	0.33
Married	0.18	0.39
Age	36.91	11.43
Employed	0.58	0.49
Unemployed	0.09	0.28
Income above £25,000	0.07	0.26
Income between £20,000-25,000	0.3	0.46
Income between £15,000-20,000	0.22	0.41
Income between £10,000-15,000	0.21	0.41
Income between £5,000-10,000	0.13	0.33
Weight (kg)	73.42	15.97
Body mass index	25.36	5.36
Blood Sugar Normal	0.9	0.3
Family History Heart Disease	0.21	0.41
Regular Diet	0.78	0.42
Vegetarian	0.15	0.36
Observations	255	

*Note*: This table provides a summary of the key characteristics of the sample used as part of this study. Means are proportions unless otherwise stated. In total, our sample consists of 255 participants, who were recruited from the surrounding precincts of the University of Edinburgh’s main campus in the city of Edinburgh. To be eligible to participate in this study, participants were required to have a good understanding of English, an annual household income below £26,500, no pre-existing medical conditions and not be pregnant. A ‘Regular Diet’ indicates a diet with no allergies or dietary restrictions i.e. not vegetarian, vegan, or, for example, free from glucose or lactose.

The first dietary measure we collected was a short-form food frequency questionnaire (FFQ), based on the US National Cancer Institute’s Dietary Screener Questionnaire. The Dietary Screener lists a total of 23 food and drink items with specific descriptors (e.g. White fish in batter or breadcrumbs—like fish and chips), and respondents are asked to indicate the frequency with which they consumed each item over the last 30 days. The FFQ contains a total of eight (8) frequency options, ranging from ‘Rarely or Never’ to ‘5+ a day’, with the exception of the seven meat and fish items (e.g. Beef, Lamb, Pork, Ham—steaks, roasts, joints, mince or chops) which had only six (6) options ranging from ‘Rarely or Never’ to ‘At least everyday’. The National Cancer Institute’s Dietary Screener Questionnaire has been subject to multiple evaluation studies, both in terms of the actual questions used as well as its overall performance and validity (e.g. [57]; [58]). A copy of the actual FFQ administered to participants is provided in Appendix A in S1 Appendix. The questionnaire was self-administered by the participants, and on average took 7 minutes to complete.

Once all participants completed the FFQ, we then moved on to the next stage of the study—a 24-hour dietary recall. For the purposes of our study, we used a web-based self-administered tool called INTAKE24, whereby users are asked to input all the food and drink items consumed over the last 24 hours. The system has been devised specifically for the UK by Newcastle University in collaboration with Food Standards Scotland, and thus contains all the major food and drink brands typically consumed by UK households, including own-brand products by the leading supermarket chains. It also features visual indicators for portion sizes, and automatically prompts users to recall items which are typically overlooked in such self-reported tools, for example snacks, sauces, side-dishes, drinks and condiments. These prompts are repeated throughout the session to minimize the risk of under-reporting. Prior to using the system, participants were asked to watch a step-by-step video tutorial showing how to utilize the INTAKE24 interface. The use of web-based dietary recalls offers various advantages over traditional face-to-face interviews, including lower costs, the possibility of larger-scale studies across different cities, more individual privacy which may lead to improved accuracy of responses due to less pressure or embarrassment, and less interviewer-specific heterogeneities which may bias results. In fact, a number of studies ([59]; [60]) have shown that self-administered online dietary recalls yield data that are statistically-comparable in terms of accuracy to those derived from face-to-face recalls. A screenshot of the INTAKE24 user interface is provided in Appendix B in S1 Appendix.

The final measure of dietary choices analyzed in this study was our own food choice tool. At the start of this intervention, all participants were allocated a budget of £30 in order to spend on the food and drink items available from our tool. £30 was chosen because the average food expenditure for the 5 first income deciles was £28.81 per person per week, based on ONS household expenditure data for 2015, (averaged taking into account both expenditure for adults and children—for details see www.gov.uk/government/statistical-data-sets/family-food-datasets and www.ons.gov.uk/peoplepopulationandcommunity/personalandhouseholdfinances/expenditure/datasets/householdexpenditurebygrossincomedecilegroupuktablea4.) Participants were allowed to spend their budget on any of the items listed in the online supermarket. Participants were instructed to spend their allocated budget with a margin of 1 pound. We did not enforce this so participants could spend less if they wish. Participants could receive up to £1 in change and were told so. For examples of where consumers were asked to spend as much of the budget as possible please see [11] and [12].

We opted for a randomized incentive scheme, whereby at the end of each of our 20 sessions, one subject per session (maximum of 18 participants per session) was drawn at random and his/her food basket was delivered to his/her home address within two weeks at his/her preferred day and time slot by a leading UK supermarket. All subjects were informed of this arrangement beforehand, including the name of the supermarket, and were reminded prior to selecting their food and drink items.

Since participants had already utilized the food choice tool as part of another experimental session in June, this meant that they were fully aware of how the incentive scheme works. Additionally, this also ensured that all participants were familiar with the tool’s user interface and were able to use it appropriately. During the June session participants were also given a mock tool containing everyday household items which they were encouraged to navigate and use beforehand in order to familiarize themselves with the actual food choice tool and its interface. To further ensure familiarity, a quick demonstration of the food choice tool was also presented to remind participants of the tool’s features.

In addition, participants were told to make their choices as though they were at their local supermarket for their weekly shop. While it is not obvious that participants will shop just for themselves these instructions can be made more specific if this is a concern to researchers using the tool. In our situation, if they were shopping for other members in the household, however, we would expect our shopping basket to be less likely to be correlated with body measurements. Below in the results section we compare the correlation between the calories and the body measurements by marital status. The idea being that single people are more likely to be purchasing just for themselves compared to those who are not single. We do not find that the differences are significant between these groups. This could suggest that the participants were either choosing for themselves or that if they were choosing for others in the household that these choices are correlated, given that meals are shared and that preferences are similar, body weight among household members are also correlated, this there might then not be surprising. Participants were allowed 10 minutes to complete their choices, with none of them requiring additional time. Once all participants had made their food and drink choices, lots were drawn to determine the winning participant and ensure that the process is as transparent and fair as possible.

Before moving onto the results it is worth stressing that the three measures of diet are not equivalent. This does not preclude comparison between the measurements nor a comparison of the measurements with weight, BMI and waist size. However, it is important to keep in mind the differences between the measures. Table 4 presents the differences in characteristics between the tools used.

**Table 4:** Comparison of the characteristics of the diet tools.

	Food choice tool	24-hour diet recall (INTAKE 24)	Food Frequency Questionnaire (short-form)
Time Period	One Week	One Day	One Month
Time-Frame	Pre-emptive	Retrospective	Retrospective
Measures	Purchases	Short-term Consumption	Long-term Consumption
Choice set	120	1560	23
Incentivized	Yes	No	No

One key difference is over what time period diet is being measured. The FFQ is attempting to capture long-run consumption, specifically over the past month. This differs compared to the food choice tool which asks participants make their shopping choice for a typical week (we try to avoid what people have in stock influencing their choices by having the basket delivered two weeks later), and the 24-hour diet recall is based on what the person ate the previous day. Therefore, if the 24-hour diet recall is a noisy measure, and the previous day to being asked to do the recall was atypical, then we might not expect to find correlation of this measurement with body size. Conversely, the longer the time period the clearer the picture of what someone eats is, so we might then expect the FFQ to be more likely correlated with body size.

Another key differentiation between the food choice tool and the FFQ and 24 hour dietary recall is that the tool is capturing purchases whereas the other two measure reported consumption. Indeed, the measures capture consumption are retrospective whereas the food choice tool is what someone might consume in the future so it is pre-emptive. The tool therefore does not actually measure what was eaten, there could of course be waste or people purchase items for others than themselves. If that is the case then this might result in the measures not being correlated but would bias the correlation between the food choice tool and the body measurements towards zero. The choice set people have also differs. INTAKE 24 is designed to have as many of the various foods people might choose, whereas the FFQ captures consumption along more broad food categories. The food choice tool has 120 popular items, however, this can be easily modified depending on the need of the researcher using it.

The final difference is that the food choice tool is the only one of the three we administer that is incentivized. The participants are making food choices and they have a chance of actually receiving these goods. We now turn to the results and investigate how each of these measures correlate with each other and then with body measurements keeping in mind that they are measuring different aspects dietary patterns.

## Results

### Summary statistics

We begin by looking at the nutrient data derived from each of our three measures. As described earlier, our proposed food choice tool contains nutritional information for each of the items displayed in the online supermarket interface, enabling us to derive the total nutrient content per basket (i.e. total calories per basket, total fats, etc.), as well as the nutrient content per £spent. Similarly, the INTAKE24 software has a database of over 1,560 food and drink items, each with a detailed breakdown of the nutrient content based on the portion size selected by the user.

The food frequency questionnaire (FFQ) is somewhat different, given that no information regarding portion sizes is recorded in the actual survey, thus complicating the conversion from frequency to nutrient intake. Nonetheless, for the purposes of our study we have developed a simple conversion protocol, inspired in part by existing methods widely used in the literature by [61] and [62].

We first calculate the average nutrient content per 100g for each of the 23 food and drink categories listed in the FFQ, based on the most popular products sold at one of the UK’s leading supermarkets for each category. The categories and food items associated with each category used in these calculations, together with their corresponding nutrient contents, are shown in Appendix C in S1 Appendix. The next step is to relate the responses from the FFQ to these nutrient values. As mentioned earlier, participants were asked to state the frequency with which they consumed items pertaining to each of the 23 categories, with 8 options ranging from ‘Rarely or Never’ to ‘5+ a day’, with the exception of the seven meat and fish items which had only six options. We therefore convert these frequency responses to estimates of **daily** intake, by assigning a numerical value to each response based on average daily consumption frequency per week. For example, if a respondent indicated that s/he consumes a particular food/drink category ‘Rarely or Never’, then her/his response is assigned a value of 0; if her/his response was ‘Once per week’, then s/he is assigned a value of 1/7 = 0.14286, reflecting the fact that on average this particular food category was only consumed once a week. We then multiply this value with the average nutrient value described above for each nutrient, to obtain an estimate of daily consumption of each nutrient.

So for example, if a subject recorded her cheese/yoghurt consumption over the last month as ‘1-2 times a day’, then this would correspond to a numerical value of 1.5, which would then be multiplied with each average nutrient for cheese/yoghurt as listed in Appendix C in S1 Appendix to derive her nutritional intake for that particular category. For example, in this case her calorie intake for cheese/yoghurt would be 1.5 × 245.5 = 368.25. Table 5 summarizes the average nutrient values of our participants from each of the three tools used in this study.

**Table 5:** Nutrient measures from each dietary assessment tool.

	Mean	Std. dev.	Min.	Max.
**A**. **Food Choice Tool (per basket)**				
Calories	10138	2899	1137	18678
Fat	252	103	10	603
Saturated Fat	87	44	2	242
Carbohydrates	1440	593	7	3183
Sugar	594	258	7	1636
Protein	478	153	116	1027
**B**. **24-hour diet recall (per day)**				
Calories	2032	1196	369	12900
Fat	83	73	1	913
Saturated Fat	31	33	0	459
Carbohydrates	240	132	18	993
Sugar	102	69	5	510
Protein	74	41	3	351
**C**. **Food Frequency Questionnaire (per day)**				
Calories	1320	662	210	5982
Fat	51	33	5	321
Saturated Fat	21	16	2	192
Carbohydrates	151	77	18	583
Sugar	63	35	11	347
Protein	56	26	9	182

*Note*: This table lists the average measurements for each nutrient, obtained from the three measures of dietary intake employed in this study, namely our own proposed food choice tool, a 24-hour dietary recall and a food frequency questionnaire (FFQ). The six nutrients measured are calories (in kcal), fat, saturated fat, carbohydrates, sugar and protein (all in grams). Both the 24-hour dietary recall and the FFQ report average nutrient intake per day, whereas our food choice tool reports the average nutrient content of each participant’s chosen food and drink basket.

### Comparison of measures

We now move on to comparing the three measures utilized in this paper in order to see how well they correlate with one another. Although we have no a priori expectation that these measures should be related to each other, given their differing data collection approaches (stated vs. revealed choices) as well as their different time period coverage, it is still nonetheless interesting to assess the extent to which they correlate with one another, given that they are derived for the same sample of participants.

Table 6 reports the pairwise correlation matrices for each of the six nutrients measured from our three measurement tools: total calories, fat, saturated fat, carbohydrates, sugar and protein. Note that whereas both the 24-hour dietary recall and the food frequency questionnaire report estimates of intake for each nutrient per day (e.g. daily calorie intake for each subject), our food choice tool only estimates the total calorie content for the entire basket of food and drink items selected, and is hence not directly comparable to the other two measures. In fact, our tool falls somewhere in between the other two, given that the 24-hour dietary recall captures food intake in a single day, and the food frequency questionnaire reflects mean daily intake based on a one-month reference point, whereas our tool covers an entire week’s worth of food choices. Nonetheless, a simple comparison at this stage serves as a useful preliminary test ahead of the main analysis in the next section, whereby the calorific data obtained from each tool will be correlated with individual biometric measures.

**Table 6:** Correlation matrices for dietary intake measures.

**(1) Calories**	Food Choice Tool	Recall	FFQ
Food Choice Tool	1		
Recall	0.06	1	
FFQ	0.05	0.05	1
**(2) Fat**	Food Choice Tool	Recall	FFQ
Food Choice Tool	1		
Recall	0.01	1	
FFQ	0.09	0.07	1
**(3) Saturated Fat**	Food Choice Tool	Recall	FFQ
Food Choice Tool	1		
Recall	0.06	1	
FFQ	0.10	0.07	1
**(4) Carbs**	Food Choice Tool	Recall	FFQ
Food Choice Tool	1		
Recall	0.13**	1	
FFQ	0.11	0.07	1
**(5) Sugar**	Food Choice Tool	Recall	FFQ
Food Choice Tool	1		
Recall	0.16**	1	
FFQ	0.21**	0.16**	1
**(6) Protein**	Food Choice Tool	Recall	FFQ
Food Choice Tool	1		
Recall	0.11	1	
FFQ	0.08	0.19**	1

*Note*: Asterisks (**) denote variables significant at 5% level. This table reports the pairwise correlations for the three measures of dietary intake used in this study, namely our proposed food choice tool, a 24-hour dietary recall and a food frequency questionnaire (FFQ), across the six nutrient measures under consideration, namely calories, fat, saturated fat, carbohydrates, sugar and protein. The idea is to assess how the measurements for each nutrient derived from each tool correlate with one another.

As seen below, the correlations across the three tools is somewhat mixed. We observe no correlations among any of the tools when it comes to calorie intake, fat and saturated fat, as seen from the first 3 panels. Nonetheless, the nutrient value for carbohydrate intake as captured by our proposed incentivized tool is correlated with the measure obtained from the the dietary recall, while the sugar intake measure is correlated with that obtained from both the 24-hour dietary recall and the FFQ. Similarly, the only correlations observed between the FFQ and the dietary recall are observed in terms of sugar and protein, which is interesting to note given that they are both self-reported measures of dietary intake. Therefore, although we do see some correlation across our proposed food choice tool and the other measures, the results also confirm the significant differences that exist between the three tools.

### Relationship between reported calorie intake and biometric indicators

We now compare the calorie measures from each tool to three common biometric indicators—weight (in kilograms), body-mass index (BMI) and waist size (in inches). The link between calorie intake and all three indicators has long been established in the literature ([14]; [63]; [64]). Therefore, a positive and statistically-significant correlation between calorie intake as captured by our tools and each indicator is expected a priori. We focus exclusively on calorie intake rather than any of the other nutrient measures since, as pointed out by [65] energy intake is the basis for one’s diet, with all other nutrients “must be provided within the quantity of food needed to fulfill the energy requirement.” Furthermore, the link between other nutrients like fat intake and each biometric indicator is not particularly robust or well-established [14], meaning that erroneous conclusions may be derived by focussing on these other nutrients.

Note that the intention here is not to determine the validity or otherwise of any of the three tools under consideration, particularly since we are only relying on a single calorie reading from each of the tools. In addition, the nutrition and epidemiology literature have used several methods to validate both the use of the FFQ as well as the 24-hour dietary recall, including cross-tool comparisons ([66]) and the use of biomarkers like 24-hour urine collections ([67]) and blood nutrient concentration ([68]). Rather, the intention in this case is to see how closely our estimates correlate with key body measurements derived from our participants, which may in turn provide some evidence to support the use of our incentivized tool as a tractable measure of dietary intake in such studies, particularly when it is infeasible to take multiple dietary readings.

We therefore estimate the following equation:

$$B_{i}=\alpha +\beta_{k}C_{k,i}+X^{\prime }\gamma_{j}+\nu_{j,i}$$ (1)

where *B*_*j*,*i*_ is the log of one of the three biometric indicators mentioned above (height, weight, BMI) for subject *i*; *C*_*k*,*i*_ is the amount of calories (in logs) pertaining to subject *i* as captured by dietary assessment tool *k*; ***X*** is a vector of control variables like age, gender, socio-economic indicators and session-specific fixed effects and *ν*_*j*,*i*_ is a random disturbance term. A complete list of control variables used in this study is provided in Appendix D in S1 Appendix.

The results are shown in Table 7, where each panel shows the results from estimating Eq 1 for each of the dietary assessment methods used in this paper (the dependent and main independent variables are in logs due to the non-normality of some of the variables and to aid the interpretation as the coefficients are elasticities). We can see that the only method whose measure of calorie intake correlates with the biometric indicators is our proposed food choice tool, as shown in Panel A. In fact, calorie intake from the food choice tool is positively and significantly-correlated with all four of the biometric indicators. The magnitudes of the coefficients of interest are somewhat low—for example a 10% increase in calories is associated with a 0.9% increase in BMI. Nonetheless, the fact that our calorie intake measure is positively correlated with each biometric measure provides further evidence to support the use of our incentivized food choice tool as a measure of people’s dietary intake. This is particularly salient given that calorie intake from neither the 24-hour dietary recall (Panel B) nor from the food frequency questionnaire (FFQ) exhibit a statistically-significant correlation with any of the indicators used, and in 4 of the 6 cases are negatively correlated. We have also estimated a model that includes all three measurement of calories in one regression. The results hold such that the strongest correlation holds calories measured with the food choice tool.

**Table 7:** Validity of dietary assessment tools.

	(1)	(2)	(3)
	Weight	BMI	Waist
**A**. **Food Choice Tool**			
Calories	0.0835**	0.0951**	0.0536*
	(0.0393)	(0.0405)	(0.0321)
Constant	3.305***	2.194***	2.841***
	(0.376)	(0.388)	(0.305)
Observations	255	255	255
R-squared	0.243	0.148	0.276
**B**. **24-hour diet recall**			
Calories	-0.0157	-0.0393	-0.0118
	(0.0323)	(0.0306)	(0.0238)
Constant	4.185***	3.354***	3.418***
	(0.296)	(0.287)	(0.223)
Observations	255	255	255
R-squared	0.229	0.133	0.267
**C**. **Food Frequency Questionnaire**			
Calories	0.0152	-0.00565	0.0102
	(0.0278)	(0.0261)	(0.0206)
Constant	3.963***	3.104***	3.261***
	(0.232)	(0.227)	(0.178)
Observations	255	255	255
R-squared	0.229	0.125	0.267

*Note*: Robust standard errors in parentheses. Asterisks (***), (**) and (*) denote variables significant at 1%, 5% and 10% levels respectively. This table reports the results from our estimates of Eq 1. In each panel, we regress the subjects’ biometric measures (weight, body-mass index and waist size, in logs) on the calorie intake values (in logs) obtained from each of the dietary intake measurement tools, namely the food choice tool, the 24-hour dietary recall and the food frequency questionnaire (FFQ), along with a number of control variables (listed in Appendix D in S1 Appendix).

There are two possible explanations for these null results: (i) a systematic misreporting of calorie intake by respondents when using both the FFQ and dietary recall, (ii) the presence of a substantial amount of noise in our data, which would raise standard errors and suppress significance levels. The first explanation is related to recent evidence on the under-reporting of calorie intake in official statistics compiled by the UK government [69]. These data, which are collated using self-reported measures of dietary intake, have shown a persistent decline in calorie intake among the general population in recent years, despite the fact that average weight and BMI has been on the rise. This corresponds to the negative (albeit not statistically-significant) coefficients we observe on our calorie intake explanatory variables in Table 7 for both the recall and the FFQ. However, we cannot discount the second explanation either, since as pointed out earlier the nutrition literature typically advocates the administration of multiple 24-hour dietary recalls in order to reduce noise within the data. In our experiment, we only consider one reading from both tools as is often the case with limited time or budgets, meaning that any conclusions regarding the reliability or otherwise of these measures must be tempered somewhat by this consideration. Additionally, the closed nature of our food choice tool with its limited range of products may also have contributed towards lowering noise levels within the data relative to the other two measures.

#### Heterogeneity in calorie reporting

We now look at how the relationship between calorie intake as measured by our 3 tools and the various biometric measures varies within different subgroups. We examine different BMI groups as well as across both genders. The aim of this exercise is to see whether any subgroups are more prone to calorie under-reporting in our data. This analysis is motivated by the fact that various studies have shown that people who are obese or overweight are more likely to under-report their dietary intake [2].

Table 8 reports quantile regression results, which looks at how the relationship between calorie intake and our biometric measures across the three tools varies according to where the respondents lie on the distribution of weight, BMI and waist measurements within our sample. As with the main analyis we are estimating models where the dependent and main independent variables are measured in logs. The analysis allows us to examine whether the correlation between measured calories and each of our anthropometrics differs across their distribution. The results show that the positive and significant relationships observed between calorie choices in our incentivized food choice tool and both BMI and waist is mainly driven by those respondents at the upper end of the distribution in each case. The esimates are less precise than those estimated at the mean (Table 2) but in general the point estimates increase the higher up the distribution. This implies that the use of incentivized tools to measure calorific intake may be particularly appropriate for eliciting truthful responses from people who are either overweight or obese—typically the same cohort who, as discussed earlier, tend to under-report in typical dietary surveys. In fact Table 6 supports the idea that those with a higher BMI under-report. Panel A shows that those with higher BMI have baskets higher in calories, i.e. they buy more. However, in panel B we see that BMI is negatively (albeit not statistically significantly so) related to the amount they report they eat.

**Table 8:** Quantile regression results.

	(1)	(2)	(3)
	Weight	BMI	Waist
**A. Food Choice Tool**			
q10	-0.0147	0.0170	0.0124
	(0.0611)	(0.0542)	(0.0567)
q25	0.0250	0.0446	0.0265
	(0.0526)	(0.0465)	(0.0480)
q50	0.0766	0.0918*	0.0266
	(0.0519)	(0.0490)	(0.0457)
q75	0.116*	0.142*	0.0534
	(0.0696)	(0.0761)	(0.0534)
q90	0.0150	0.133	0.0929*
	(0.0869)	(0.0942)	(0.0531)
**B**. **24-hour diet recall**			
q10	-0.0206	-0.0271	-0.0215
	(0.0435)	(0.0377)	(0.0398)
q25	-0.0450	-0.0281	0.00106
	(0.0459)	(0.0443)	(0.0333)
q50	0.00232	-0.0366	-0.0135
	(0.0463)	(0.0383)	(0.0315)
q75	0.0301	-0.0244	-0.00926
	(0.0464)	(0.0459)	(0.0376)
q90	0.0105	0.00541	-0.00271
	(0.0581)	(0.0521)	(0.0507)
**C. Food Frequency Questionnaire**			
q10	0.0477	-0.0205	0.0637*
	(0.0435)	(0.0388)	(0.0324)
q25	0.0147	0.00538	0.0278
	(0.0439)	(0.0382)	(0.0295)
q50	0.00375	-0.0128	0.00699
	(0.0406)	(0.0307)	(0.0340)
q75	0.0104	-0.0352	-0.0145
	(0.0426)	(0.0451)	(0.0387)
q90	-0.00760	-0.0349	0.0138
	(0.0524)	(0.0489)	(0.0449)
Controls	Y	Y	Y
Observations	255	255	255

*Note*: Robust standard errors in parentheses. Asterisks (***), (**) and (*) denote statistical significance at 1%, 5% and 10% levels respectively. This table reports the results from our quantile regression estimates of Eq 1, where we consider different percentiles of the distribution of each of our dependent variables (e.g. Q10 represents the 10th percentile, Q90 represents the 90th percentile, etc.). The idea behind this analysis is to assess whether the results presented in 7 differ among subjects according to where they are on the distribution of weight, BMI and waist within the sample. In each panel, we once again regress the subjects’ biometric measures (weight, body-mass index and waist size, in logs) on the calorie intake values (in logs) obtained from each of the dietary intake measurement tools, namely the food choice tool, the 24 hour dietary recall and the food frequency questionnaire (FFQ), along with a number of control variables (listed in Appendix D in S1 Appendix).

There are a number of potential reasons for these results. First, this could be due to there being greater dispersion in BMI and waist at higher levels of each measure. More specifically, the standard deviation for people with a BMI of 25 and above the standard deviation is 5.08, and below 25 is 1.85; and the standard deviation for those whose waist is above average is 4.44, falling to 2.42 for respondents below the average. Therefore, the observed relationships may simply reflect the statistical properties of our sample, and in particular the lack of dispersion in the below average waist group, and the sub-25 BMI group. Nonetheless, there are other potential explanations for these findings. For example, [70] find a positive and significant correlation between TV viewing/screen time and calorie intake among children. However, this positive relationship is particularly prevalent among overweight or obese children, thus implying that the association between dietary intake and biometric measurements is stronger at higher levels of each measure. In addition, studies on physical activity show that overweight or obese people participate significantly less in such activities relative to normal weight individuals ([71]; [72]), which in turn may account for the lack of correlation that we observe between calorie intake and BMI among this cohort [73]. Therefore, the combination of differences in dispersion within our dataset, as well as differences in physical activity across normal and overweight individuals, may be contributing to the statistically-significant relationship between calorie intake and anthropometric measures among overweight/obese participants in our tool, and on the flipside the lack of correlation among normal or lower weight participants.

In Table 9 we perform another subgroup analysis, only this time analyzing differences according to gender. None of our estimates from any of our dietary measurement tools are statistically-significant for male participants (columns 1—3). However, we do find positive and significant results for our female subgroup (columns 4—6) when using our proposed food choice tool. Thus, it appears as though using incentivized tools for capturing dietary measures may be more effective for female participants rather than males, while self-reported measures are equally ineffective for both genders.

**Table 9:** Subgroup analysis—By gender.

	(1)	(2)	(3)	(4)	(5)	(6)
	Male			Female		
	Weight	BMI	Waist	Weight	BMI	Waist
**A**. **Food Choice Tool**						
Calories	-0.0277	-0.0450	-0.0402	0.128**	0.140***	0.0806*
	(0.0588)	(0.0532)	(0.0404)	(0.0493)	(0.0485)	(0.0434)
Constant	4.318***	3.325***	3.695***	2.989***	1.884***	2.674***
	(0.607)	(0.530)	(0.407)	(0.456)	(0.458)	(0.404)
Observations	96	96	96	159	159	159
R-squared	0.315	0.314	0.323	0.170	0.223	0.290
**B**. **24-hour diet recall**						
Calories	-0.0217	-0.0604	-0.0271	-0.00195	-0.0163	-0.000659
	(0.0495)	(0.0487)	(0.0305)	(0.0527)	(0.0479)	(0.0389)
Constant	4.227***	3.383***	3.529***	4.165***	3.276***	3.410***
	(0.478)	(0.470)	(0.286)	(0.439)	(0.413)	(0.327)
Observations	96	96	96	159	159	159
R-squared	0.316	0.331	0.322	0.137	0.183	0.270
**C**. **Food Frequency Questionnaire**						
Calories	-0.0289	-0.0510	-0.00160	0.0416	0.0236	0.0207
	(0.0373)	(0.0341)	(0.0318)	(0.0408)	(0.0376)	(0.0275)
Constant	4.250***	3.244***	3.321***	3.867***	2.997***	3.264***
	(0.312)	(0.295)	(0.259)	(0.320)	(0.309)	(0.235)
Observations	96	96	96	159	159	159
R-squared	0.318	0.325	0.314	0.144	0.185	0.273

*Note*: Robust standard errors in parentheses. Asterisks (***), (**) and (*) denote variables significant at 1%, 5% and 10% levels respectively. This table reports the results from our linear regression estimates of Eq 1, only this time focusing solely on male subjects. The idea behind this subgroup analysis is to assess whether the results presented in Table 7 differ among men. In each panel, we once again regress the subjects’ biometric measures (weight, body-mass index and waist size) on the calorie intake values obtained from each of the dietary intake measurement tools, namely the food choice tool, the 24 hour dietary recall and the food frequency questionnaire (FFQ), along with a number of control variables (listed in Appendix D in S1 Appendix).

Table 10 examines the differences by marital status and household size. In particular, we first estimate (in columns 1—3) the correlation between the body measurements and the food choice tool for those who are married or are in households of more than one person. In the right-hand side of the table (columns 4—6) we examine those who have 1 person in the household or are single. The correlations are only statistically significant for our incentivised supermarket tool. We find that correlation is larger and significant for the correlation between calories and BMI, however the difference between the two groups are not statistically significant. As before we do not find any significant correlations between the body measurements and the other methods of diet elicitation.

**Table 10:** Subgroup analysis—By marital status & household size.

	(1)	(2)	(3)	(4)	(5)	(6)
	Married or Household>1			Not married or household = 1		
	Weight	BMI	Waist	Weight	BMI	Waist
**A**. **Food Choice Tool**						
Calories	0.0894*	0.0993**	0.0544	0.0224	0.0219	0.0427
	(0.0469)	(0.0470)	(0.0367)	(0.0804)	(0.0764)	(0.0628)
Constant	3.296***	2.210***	2.891***	4.013***	3.038***	2.991***
	(0.457)	(0.456)	(0.363)	(0.764)	(0.746)	(0.597)
Observations	178	178	178	77	77	77
R-squared	0.238	0.158	0.238	0.419	0.389	0.544
**B**. **24-hour diet recall**						
Calories	-0.00679	-0.0298	0.00145	-0.0241	-0.0415	-0.0294
	(0.0418)	(0.0420)	(0.0318)	(0.0609)	(0.0517)	(0.0406)
Constant	4.179***	3.350***	3.389***	4.390***	3.541***	3.585***
	(0.353)	(0.359)	(0.286)	(0.629)	(0.528)	(0.423)
Observations	178	178	178	77	77	77
R-squared	0.220	0.135	0.227	0.421	0.398	0.547
**C**. **Food Frequency Questionnaire**						
Calories	0.00552	-0.0196	0.00495	0.0383	0.0305	0.00878
	(0.0370)	(0.0343)	(0.0282)	(0.0721)	(0.0640)	(0.0468)
Constant	4.092***	3.271***	3.365***	3.901***	2.984***	3.295***
	(0.291)	(0.274)	(0.229)	(0.650)	(0.581)	(0.451)
Observations	178	178	178	77	77	77
R-squared	0.220	0.133	0.227	0.423	0.392	0.541

*Note*: Robust standard errors in parentheses. Asterisks (***), (**) and (*) denote variables significant at 1%, 5% and 10% levels respectively. This table reports the results from our linear regression estimates of Eq 1. In each panel, we regress the subjects’ biometric measures (weight, body-mass index and waist size, in logs) on the calorie intake values (in logs) obtained from each of the dietary intake measurement tools, namely the food choice tool, the 24 hour dietary recall and the food frequency questionnaire (FFQ), along with a number of control variables (listed in Appendix D in S1 Appendix).

### Sensitivity of dietary selection in food choice tool

Having looked at our incentivized food choice tool as a predictor of people’s biometric measurements and dietary intake, we now turn to assessing its functionality. More specifically, in this section we analyze the sensitivity of people’s food choices to changes in the food/drink category first shown to participants upon logging into the system. In order to access the food choice tool, participants in the experiment were first asked to type in their username, after which the tool was loaded and they were immediately shown one of the six categories of food and drink items mentioned earlier, namely fruit and vegetables, meat and fish, bread and grains, confectionery and snacks, ready meals and drinks. The category displayed onscreen varied randomly across participants in order to avoid any systematic priming effects, and obviously participants were free to browse any of the other categories once the system loaded. Nonetheless, it is possible to assess whether initial exposure to any of these categories had any significant impact on both the type of food selected, as well as the nutrient content of their chosen food baskets. This is an important consideration, and is related to the substantial literature on mindless food choices and how people tend to opt for those food and drink items that are easily accessible, in various settings from supermarkets to restaurants and cafeterias ([74]; [10]; [75]). Thus, initial food category exposure may have some impact on participants’ food choices in our tool, and must be taken into account and controlled for when conducting experiments or other studies using this tool.

Table 11 summarizes participants’ initial exposure to each supermarket category in our food choice tool. As seen below, the initial category shown to participants upon login was fairly evenly-distributed across the six categories.

**Table 11:** Initial category exposure in food choice tool.

Variable	Mean	Std. Dev.
Fruit and Veg	12.9%	33.6
Meat and Fish	17.3%	37.9
Bread and Grains	18%	38.5
Confectionery	17.6%	38.2
Ready Meals	16.1%	36.8
Drinks	18%	38.5
N	255	

*Note*: This table shows the proportion of subjects who, upon accessing the food choice tool, were initially exposed to each of the six food and drink categories available, namely fruit and vegetables, meat and fish, bread and grains, confectionery and snacks, ready meals and drinks.

We can now formally assess whether initial exposure to any one of these 6 categories had any impact on participants’ food and drink choices. We estimate the following equation:

$$Y_{ji}=\alpha +\sum_{k=1}^{5}\beta_{ki}Front_{ki}+{X_{i}}^{\prime }\gamma +\epsilon_{i};\forall j\neq k$$ (2)

where *Y*_*ji*_ is our dependent variable, which in this case will be either total expenditure on each of the *j* = {1, 2, 3, 4, 5, 6} supermarket categories by subject *i*, or the total number of items selected per category by *i*. Our explanatory variables of interest are represented by (6-1) five dummy variables *Front*_*ki*_, whereby each dummy takes a value of 1 if subject *i* was first exposed to that particular food/drink category when using our food choice tool. To facilitate the interpretation of our results, we use the same baseline (omitted) category as the one used in our dependent variable. Thus for example, if we consider total expenditure on fruit and vegetables as our dependent variable, then the baseline or omitted category for our initial exposure dummies will be the fruit and vegetables category. The rationale behind this specification is that if initial exposure to a particular category led to increased expenditure on items pertaining to that same category, then some of the coefficients on the other category dummies should be negative and statistically-significant. ***X***_***i***_ is the same vector of control variables used in Table 7, with a full list of variables provided in Appendix D in S1 Appendix.

Table 12 presents our findings. Panel A shows the results obtained when using total expenditure on each category as our dependent variable; Panel B uses the total number of items selected per category while As seen below, the results are somewhat mixed. We start with Panel A, column 1, where we observe negative and significant coefficients on both the confectionery and drinks categories, indicating that initial exposure to the fruit and veg category led to higher expenditure on fruit and veg relative to when the initial category was confectionery and drinks, to the tune of £3.17 and £2.39 respectively. The coefficients on the other categories are also negative, albeit not precisely estimated. In column 4 we obtain a statistically-significant coefficient on ready meals and on the drinks category. The rest of the results in the remaining columns are largely insignificant, indicating that on the whole there does not seem to be any systematic effect of initial category exposure on food expenditure.

**Table 12:** Sensitivity of food choices to initial category exposure.

	(1)	(2)	(3)	(4)	(5)	(6)
	**Fruit**	**Meat**	**Bread**	**Confectionery**	**Ready Meals**	**Drinks**
**A**. **Expenditure per Category**						
**Front page**						
Fruit		-0.742	0.283	-0.524	-0.00978	-0.306
		(1.494)	(0.617)	(1.056)	(0.567)	(0.550)
Meat	-0.869		-0.518	-1.014	0.932	0.111
	(1.111)		(0.562)	(0.712)	(0.630)	(0.469)
Bread	-0.881	-1.765		-1.133	0.689	0.106
	(1.186)	(1.293)		(0.761)	(0.558)	(0.463)
Confectionery	-3.173**	0.101	-0.123		0.935*	-0.200
	(1.225)	(1.340)	(0.601)		(0.540)	(0.465)
Ready Meals	-1.747	1.243	-0.486	-2.078***		-0.497
	(1.153)	(1.229)	(0.507)	(0.712)		(0.529)
Drinks	-2.389**	1.400	-0.737	-1.981**	0.459	
	(1.126)	(1.181)	(0.537)	(0.764)	(0.736)	
Constant	13.93***	10.76***	4.283**	-0.713	4.575***	1.839
	(2.904)	(3.636)	(1.683)	(2.215)	(1.515)	(1.302)
	**Fruit**	**Meat**	**Bread**	**Confectionery**	**Ready Meals**	**Drinks**
**B**. **Items chosen per category**						
**Front page**						
Fruit		0.0351	0.315	-0.606**	-0.376	-0.0448
		(0.512)	(0.451)	(0.285)	(0.303)	(0.270)
Meat	-1.003		-0.445	-0.147	-0.136	0.247
	(0.787)		(0.357)	(0.263)	(0.283)	(0.295)
Bread	-0.849	-0.330		0.327	0.0973	0.143
	(0.905)	(0.403)		(0.307)	(0.269)	(0.267)
Confectionery	-2.364**	0.528	0.0109		-0.254	0.324
	(0.936)	(0.438)	(0.414)		(0.276)	(0.249)
Ready Meals	-1.751**	0.0963	-0.748**	-0.309		0.603
	(0.845)	(0.435)	(0.347)	(0.291)		(0.446)
Drinks	-1.633**	0.824*	-0.723**	-0.144	-0.179	
	(0.819)	(0.428)	(0.332)	(0.282)	(0.284)	
Constant	11.71***	4.015***	1.632	0.578	0.338	0.573
	(2.146)	(1.168)	(1.034)	(0.739)	(0.696)	(0.850)

*Note*: Robust standard errors in parentheses. Asterisks (***), (**) and (*) denote variables significant at 1%, 5% and 10% levels respectively. This table reports the results from our linear regression estimates of Eq 2. Panel A regresses total expenditure on each food and drink category (in £) on a series of dummy variables denoting which category the subjects were initially exposed to upon accessing the food choice tool. In Panel B a similar regression is carried out, only this time our dependent variable in each column is the total number of items per category selected by each subject. Note that in both Panels A and B the omitted category in each column corresponds to the category used as the dependent variable, in order to facilitate the interpretation of results. For example, in Panel A, Column 1, since our dependent variable is total expenditure on fruit and vegetables, the omitted (dummy) initial category is also fruit and vegetables.

Similarly mixed results are observed in Panel B, where we consider the total number of items selected in each category as our dependent variables. Once again we observe negative and significant coefficients in column 1 for confectionery, drinks and also ready meals, which provides some evidence that in this case initial exposure to fruit and veg did indeed increase subject’s fruit and veg choices relative to the other categories. We also observe some systematic impact when it comes to bread and grains (column 3), where we have negative and significant coefficients on both confectionery and drinks, although the other category coefficients are not significant. Nonetheless, as before we also find a positive coefficient on the drinks category in column 2 in relation to meat and fish, which would seemingly indicate that people exposed to the drinks category selected more meat items that participants whose initial category was meat and fish, contrary to expectations. Thus, although in this instance there is some evidence of systematic bias in the choices of participants based on which category they were initially exposed to (especially with regards to fruit and veg), overall this does not seem to be the case for most of our food and drink categories. Therefore, the pattern that emerges here is that although varying the initial category exposure in our food choice tool can have a significant impact on actual choices, there is limited evidence of this systematically nudging or boosting choices within the same category of exposure. Thus, the results discussed in Table 12 highlight the importance of taking this initial category exposure into account in any analysis that makes use of our proposed incentivized food choice tool.

A final point is related to the possibility that our food choice tool may be capturing expenditure on luxury items or treats, particularly if they considered this shopping trip to be a bonus over and above their typical weekly shop. In the instructions to the participants we state:“We kindly ask you to make your food and drink choices carefully as though you were at your local supermarket for your weekly shop.” Of course, we cannot prevent the participants choosing whatever they want and selecting just luxuries and treats. There is a trade-off here: we think the incentivised measure increases the probability that someone chooses a basket of goods that they eat, and if we did not incentivise we would be worried that they would not do so. If a researcher is concerned about this they could easily limit the number of times an item was chosen, or remove “luxury” items from the tool whatsoever—this in turn has its own disadvantages. We chose to allow participants a free-hand in what they chose but asked them to make their choices as though they were at their local supermarket for their weekly shop. Nonetheless, it is worth probing a little further in order to analyse participants’ actual food choices. To this end, we have examined the participants’ choices of “luxury” items like treats. On average only 7.1% of total budget was spent on confectionery items, which include treats like sweets and chocolates; the highest recorded in our sample was 32.8%. Therefore, it does not seem as though our subjects opted to spend their budget on treats. The highest proportion of participants’ budget was spent on meat and fish (37%). Meat items were, on average, the most expensive products in our supermarket with an average price per item of £3.06. Therefore, an argument could be made that meat and fish were ‘luxury’ items within our supermarket, and that people gravitated towards these items due to windfall gains. However, the 37% spent on meat and fish is close to the average weekly expenditure by UK households, which is approximately 35% as per ONS Family Spending Data [76]. Therefore, it appears that the participants’ food choices largely mirrored those that they typically make at the supermarket on a weekly basis, in line with our instructions, and that there was no evident focus on purchasing luxury items or treats.

## Concluding remarks

In this paper, we present and evaluate a new low-cost tool for eliciting information about food choices in an incentivized manner. People are allocated a fixed budget to spend on various food and drinks items from across six different categories, namely fruit and vegetables, meat and fish, bread and grains, confectionery and snacks, ready meals and drinks. The proposed food choice tool has several important benefits. Firstly, our tool can gather information regarding people’s food choices in a relatively quick and inexpensive manner, without having to resort to multiple readings as with other tools used by nutritionists and epidemiologists. Secondly, the inherent flexibility of the tool means that it can be adjusted and tailored to suit a wide variety of research needs, and can be administered in various settings, even online. Thirdly, since our tool is designed to mimic an online supermarket, it captures a relatively wide variety of foods and drinks, encompassing a number of meals and snacks, thus enabling a more representative picture of people’s dietary choices to be formulated. Finally, by incentivizing people’s responses via the possibility of receiving their chosen food basket, this tool is able to avoid some of the pitfalls commonly associated with self-reported measures of dietary intake, particularly when it comes to misreporting [77].

To test out the functionality of our new tool, we conducted a lab experiment among 255 real-world participants at the University of Edinburgh’s BLUE lab, whereby participants’ dietary intake was recorded using three different tools, namely a food frequency questionnaire, a 24-hour dietary recall and our own food choice tool. Both the food frequency questionnaire and the dietary recall are heavily-used in the nutrition and epidemiology literature and have been validated, although both tools must be administered more than once in order to obtain more representative estimates of dietary intake. In addition, their reliability in terms of the representativeness of the measurements obtained has been called into question recently due to the fact that they both rely on self-reported dietary intake [2].

We first sought to compare the values for nutrient profile across each of the three tools to see how well they match up to one another. The results were somewhat mixed, although no statistically-significant correlation was observed across the tools when considering calories, fat and saturated fat. We then proceeded to compare the total calorie intake recorded for each subject by the three tools to a number of individual biometric measures which are known to be positively-correlated with calorie intake, namely weight, body mass index (BMI) and waist size. The results showed that only the calorie intake measure from our incentivized food choice tool was positively and significantly related to each of the four biometric measures. By contrast, the calorie measures obtained from both the food frequency questionnaire and the 24 hour dietary recall were not significantly related to *any* of the biometric measures under consideration. Analysis of different subgroups within our sample showed that the positive and significant correlation observed for our incentivized tool was mainly driven by overweight or obese participants, which must be seen in light of recent evidence on under-estimation of calorie intake among this cohort when using self-reported measurement tools like a food frequency questionnaire or a 24 hour dietary recall [69].

Finally, we examined whether our food choice tool was sensitive to particular design choices, specifically by assessing whether variation in the initial category of food that participants were exposed to upon accessing the tool had any impact on their actual choices. The results were somewhat scattered, with limited evidence that initial exposure had any impact on encouraging greater purchases from that same category, although we did find some statistically-significant impacts across categories. Thus, these findings showed the importance of adequately controlling for initial category exposure when conducting any work based on this tool.

The findings in this paper provide further evidence that incentive-based tools for information elicitation can provide significant benefits in terms of capturing people’s underlying preferences and behaviours, particularly when financial resources are limited and quick, one-shot dietary readings are required without repetition. Amidst growing concern regarding self-reported data in surveys, this study highlights the possibility of integrating novel techniques that incentivize truthful responses into standard surveys across a wide range of applications, while keeping issues related to cost and practicality in check. This paper also lends further credence to the concerns raised in [39] and [69] regarding the systematic biases in self-reported survey responses, and thus the need to control for such errors when conducting any empirical investigation.

## Supporting information

S1 Appendix (PDF)
