# Supplementary material for: A computer-based incentivized food basket choice tool: Presentation and evaluation
Source: PLoS One. 2019 Jan 10;14(1):e0210061. doi: 10.1371/journal.pone.0210061 (PMC6328152; doi:10.1371/journal.pone.0210061)
Supplement: S1 Appendix — (PDF) [file pone.0210061.s001.pdf]

Appendices

Appendix A: Copy of Food Frequency Questionnaire

These questions are about food and drink you consumed during the past month, that is, the past 30 days. When answering, please include meals and snacks at home, at work, in restaurants, or anywhere else.

Please enter your username

Please enter your desk number

Please tick how often you ate at least ONE portion of the following foods and drinks over the past month (a portion includes a handful of grapes, an orange, a serving of carrots, a side salad, a slice of bread, a glass of liquid).

	Rarely or Never	Less than 1 a Week	Once a week	2-3 times a week	4-6 times a week	1-2 times a day	3-4 times a day	5+ a day
Fruit (tinned/fresh)	○	○	○	○	○	○	○	○
Fruit juice (not cordial or squash)	○	○	○	○	○	○	○	○
Salad (not garnish added to sandwiches)	○	○	○	○	○	○	○	○
Vegetables (tinned / frozen / fresh but not potatoes	○	○	○	○	○	○	○	○
Chips / fried potatoes	○	○	○	○	○	○	○	○
White rice or potatoes								

like baked, boiled, mashed or sweet potatoes	O	O	O	O	O	O	O	O
Beans or pulses like baked beans, lentils, chick peas, dahl	O	O	O	O	O	O	O	O
Tea or coffee sweetened with sugar or honey, including iced tea and frappuccino	O	O	O	O	O	O	O	O

Please tick how often you ate at least ONE portion of the following foods and drinks over the past month (a portion includes a 30g bowl of cereal, 25g of cheese, a packet of crisps, 2 biscuits, a slice of bread, a glass of liquid).

	Rarely or Never	Less than 1 a Week	Once a week	2-3 times a week	4-6 times a week	1-2 times a day	3-4 times a day	5+ a day
Fibre-rich breakfast cereal like Weetabix, Fruit 'n Fibre, Porridge, Muesli	O	O	O	O	O	O	O	O
Wholemeal bread or chapattis	O	O	O	O	O	O	O	O
Brown rice / bulgur wheat / quinoa	O	O	O	O	O	O	O	O
Cheese / yoghurt	O	O	O	O	O	O	O	O
Crisps / savoury snacks	O	O	O	O	O	O	O	O
Sweet biscuits, cakes, chocolate, sweets	O	O	O	O	O	O	O	O
Ice cream / cream	O	O	O	O	O	O	O	O
Non alcoholic fizzy drinks / pop (not sugar free or diet)	O	O	O	O	O	O	O	O

Please tick how often you ate at least ONE portion of the following foods and drinks over the past month.

	Rarely or Never	Less than 1 a Week	Once a week	2-3 times a week	4-6 times a week	At least everyday
Beef, Lamb, Pork, Ham - steaks, roasts, joints,	O	O	O	O	O	O

mince or chops						
Chicken or Turkey - steaks, roasts, joints, mince or portions (not in batter or breadcrumbs)	O	O	O	O	O	O
Sausages, bacon, corned beef, meat pies/pasties, burgers	O	O	O	O	O	O
Chicken/turkey nuggets. turkey burgers, chicken pies, or in batter or breadcrumbs	O	O	O	O	O	O
White fish in batter or breadcrumbs - like fish 'n chips	O	O	O	O	O	O
White fish not in batter or breadcrumbs	O	O	O	O	O	O
Oily fish - like herrings, trout, salmon, sardines, mackerel, fresh tuna (not tinned tuna)	O	O	O	O	O	O

What milk do you USUALLY use or drink, such as in hot & cold drinks or on cereal (including tea, coffee, hot milk, milkshakes, or on cereal)

Whole / full-fat milk

Semi-skimmed milk

Skimmed Milk

Rarely / never use milk

Other (please specify)

Appendix B: Screenshot of 24-Hour Dietary Recall (INTAKE24)

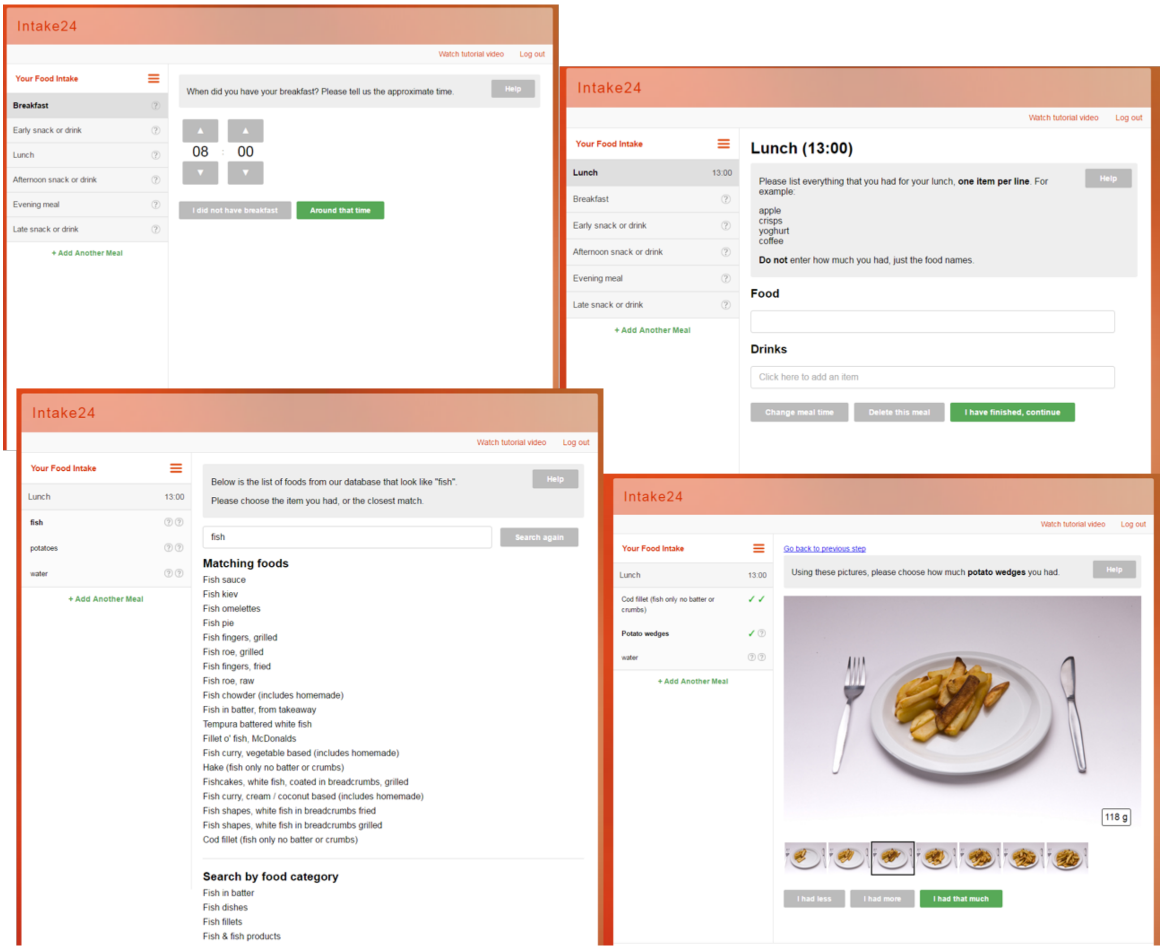

Appendix C: Food and Drink Items used in Food Frequency Questionnaire Nutrient Calculations

Category	Item
Fruit	Royal Gala Apples
Fruit	Fairtrade Bananas
Fruit	Easy Peeler Oranges
Fruit	Red Seedless Grapes
Fruit	Conference Pears
Fruit	Raspberries
Fruit	Strawberries
Fruit	Peaches
Fruit	Kiwi Fruit
Fruit juice	Smooth Orange Juice, Not from Concentrate
Fruit juice	Smooth Orange Juice, from Concentrate
Fruit juice	Pressed Apple Juice, Not from Concentrate
Fruit juice	Apple Juice, From Concentrate
Fruit juice	Orange Juice with Bits, Not From Concentrate
Fruit juice	Apple and Mango Juice, Not from Concentrate
Fruit juice	Mango and Passion Fruit Smoothie
Fruit juice	Strawberry and Banana Smoothie
Salad	Bistro Salad
Salad	Italian Style Salad
Salad	Mixed Leaf Salad
Salad	Sweet Leaf Salad
Salad	Babyleaf Salad
Salad	Crispy Salad
Vegetables	Cherry Tomatoes
Vegetables	Closed Cup White Mushrooms
Vegetables	Mixed Peppers
Vegetables	Carrots
Vegetables	Onions
Vegetables	Fine Beans
Vegetables	Broccoli
Vegetables	Sweetcorn
Vegetables	Cucumber
Vegetables	Red Onions
Chips/fried potatoes	Homestyle Chips
Chips/fried potatoes	Straight Cut Chips
Chips/fried potatoes	French Fries
Chips/fried potatoes	Oven Chips
White rice or potatoes	Baked Potatoes
White rice or potatoes	Maris Piper Potatoes
White rice or potatoes	Baby Potatoes
White rice or potatoes	Sweet Potatoes
White rice or potatoes	Mashed Potatoes
White rice or potatoes	Basmati Rice
White rice or potatoes	Arborio Rice
Beans or pulses	Baked Beans
Beans or pulses	Baked Beans in Tomato Sauce
Beans or pulses	Green Lentils
Beans or pulses	Red Lentils
Beans or pulses	Chickpeas

Category	Item
Tea or coffee sweetened	Tea
Tea or coffee sweetened	Capuccino
Tea or coffee sweetened	Barista Coffee
Fibre-rich breakfast cereal	Weetabix
Fibre-rich breakfast cereal	Fruit and Fibre
Fibre-rich breakfast cereal	Porridge
Fibre-rich breakfast cereal	Muesli
Wholemeal bread or chapattis	Wholemeal Bread
Brown rice/bulgur wheat/quinoa	Brown Basmati Rice
Brown rice/bulgur wheat/quinoa	Quinoa
Brown rice/bulgur wheat/quinoa	Bulgur Wheat
Cheese/yoghurt	Mature Cheddar
Cheese/yoghurt	Red Leicester
Cheese/yoghurt	Greek Style Yoghurt
Cheese/yoghurt	Natural Yoghurt
Crisps/savoury snacks	Tortilla Crisps
Crisps/savoury snacks	Cheese and Onion Crisps
Crisps/savoury snacks	Salted Crisps
Crisps/savoury snacks	Cashews
Crisps/savoury snacks	Peanuts
Sweet biscuits, cakes, chocolate, sweets	Milk chocolate digestives
Sweet biscuits, cakes, chocolate, sweets	Rich Tea Biscuits
Sweet biscuits, cakes, chocolate, sweets	Shortbread
Sweet biscuits, cakes, chocolate, sweets	Pain au Chocolat
Sweet biscuits, cakes, chocolate, sweets	Chocolate Cake
Sweet biscuits, cakes, chocolate, sweets	Milk Chocolate Fingers
Sweet biscuits, cakes, chocolate, sweets	Chocolate Buttons
Sweet biscuits, cakes, chocolate, sweets	Fudge Chocolate Bar
Sweet biscuits, cakes, chocolate, sweets	Assorted Candy
Sweet biscuits, cakes, chocolate, sweets	Marshmallows
Ice cream/cream	Vanilla Ice Cream
Ice cream/cream	Ice Cream Cone
Ice cream/cream	Whipped Cream
Non alcoholic sugary fizzy drinks/pop	Cola
Non alcoholic sugary fizzy drinks/pop	Lemonade
Non alcoholic sugary fizzy drinks/pop	Orange Ade
Non alcoholic sugary fizzy drinks/pop	Irn Bru
Beef, lamb, pork, ham	Beef Mince
Beef, lamb, pork, ham	Honey Roast Ham
Beef, lamb, pork, ham	Beef Joint
Beef, lamb, pork, ham	Beef Steak
Beef, lamb, pork, ham	Gammon Joint
Beef, lamb, pork, ham	Pork Loin
Beef, lamb, pork, ham	Pork Chops
Beef, lamb, pork, ham	Lamb Mince
Beef, lamb, pork, ham	Lamb Leg
Beef, lamb, pork, ham	Lamb Chops
Chicken or turkey	Chicken Breats Fillets
Chicken or turkey	Chicken Thigh Fillets
Chicken or turkey	Whole Chicken
Chicken or turkey	Turkey Mince
Chicken or turkey	Turkey Breast Steaks
Chicken or turkey	Turkey Breast Fillets
Sausages, bacon, meat pies, burgers	Unsmoked Bacon
Sausages, bacon, meat pies, burgers	Pork Sausages
Sausages, bacon, meat pies, burgers	Smoked Bacon
Sausages, bacon, meat pies, burgers	Cumberland Sausages
Sausages, bacon, meat pies, burgers	Beef Burgers
Sausages, bacon, meat pies, burgers	Beef Meatballs

Category	Item
Sausages, bacon, meat pies, burgers	Pork Pie
Sausages, bacon, meat pies, burgers	Steak Pie
Chicken/turkey nuggets, burgers, pies	Chicken Kiev Garlic
Chicken/turkey nuggets, burgers, pies	Chicken Kiev Ham
Chicken/turkey nuggets, burgers, pies	Chicken Nuggets
Chicken/turkey nuggets, burgers, pies	Chicken Pie
Chicken/turkey nuggets, burgers, pies	Turkey Escalope
White fish in batter or breadcrumbs	Haddock Fishcakes
White fish in batter or breadcrumbs	Breaded Cod
White fish in batter or breadcrumbs	Cod Fishcakes
White fish in batter or breadcrumbs	Dusted Lemon Sole
White fish not in batter or breadcrumbs	Cod Fillets
White fish not in batter or breadcrumbs	Sea Bass Fillets
White fish not in batter or breadcrumbs	Basa Fillets
White fish not in batter or breadcrumbs	Plaice Fillets
Oily fish (not tinned)	Salmon Fillet
Oily fish (not tinned)	Smoked Salmon Fillet
Oily fish (not tinned)	Trout Fillet
Oily fish (not tinned)	Mackerel Fillets

Category	Calories	Fat	Sat Fat	Carbohydrates	Sugar	Protein
Fruit (tinned/fresh)	49.444	0.433	0.089	10.944	10.578	0.878
Fruit juice	44.750	4.688	3.938	9.925	8.900	0.538
Salad	20.667	0.467	0.100	1.733	1.283	1.717
Vegetables	45.100	0.630	0.100	4.630	3.310	1.630
Chips/fried potatoes	199.500	5.975	0.725	32.400	0.500	2.925
White rice or potatoes	109.714	1.157	0.457	21.886	3.443	2.429
Beans or pulses	89.000	0.720	0.140	12.400	2.200	5.920
Tea or coffee sweetened	19.333	0.433	0.367	3.167	3.633	0.567
Fibre-rich breakfast cereal	369.750	5.100	1.425	66.000	11.975	10.650
Wholemeal bread or chapattis	221.000	1.800	0.400	37.800	4.100	10.000
Brown rice/bulgur wheat/quinoa	112.000	1.800	0.500	19.033	0.400	3.700
Cheese/yoghurt	245.500	19.350	12.575	3.200	3.150	14.925
Crisps/savoury snacks	555.000	38.520	5.080	36.720	3.500	13.160
Sweet biscuits, cakes, chocolate, sweets	445.700	18.310	9.510	63.990	41.010	5.440
Ice cream/cream	284.333	19.467	14.233	24.100	17.767	2.667
Non alcoholic sugary fizzy drinks	35.250	0.000	0.000	8.600	8.600	0.000
Beef, lamb, pork, ham	218.700	12.260	5.230	0.970	0.590	26.200
Chicken or turkey	161.500	4.117	1.083	0.500	0.500	30.583
Sausages, bacon, meat pies, burgers	244.375	14.663	5.638	9.075	1.350	18.550
Chicken/turkey nuggets, burgers, pies	262.400	15.480	3.980	17.040	1.260	13.140
White fish in batter or breadcrumbs	191.500	8.475	1.625	15.175	1.025	13.100
White fish not in batter or breadcrumbs	129.000	4.200	1.025	0.500	0.500	22.625
Oily fish (not tinned)	217.500	14.550	3.075	0.675	0.500	21.150

Appendix D: List of Control Variables

Variable Name	Description of Variable
Male	Dummy denoting whether subject is male or not
Age	Variable denoting the subject's stated age
Employed	Dummy denoting whether subject is currently gainfully-occupied
Unemployed	Dummy denoting whether subject is currently unemployed and seeking work
Student	Dummy denoting whether subject is currently a full-time student
Postgrad	Dummy denoting whether subject's highest level of education is a post-graduate degree
Undergrad	Dummy denoting whether subject's highest level of education is an under-graduate degree
A-level	Dummy denoting whether subject's highest level of education is an A-level certificate (or equivalent)
Income > £25000	Dummy denoting whether subject's annual household income is above £25,000
Income £20000-£25000	Dummy denoting whether subject's annual household income is between £20,000 and £25,000
Income £15000-£20000	Dummy denoting whether subject's annual household income is between £15,000 and £20,000
Income £10000-£15000	Dummy denoting whether subject's annual household income is between £10,000 and £15,000
Income £5000-£10000	Dummy denoting whether subject's annual household income is between £5,000 and £10,000
White	Dummy denoting whether subject's race is Caucasian
Married	Dummy denoting whether subject is currently married
Blood Sugar	Dummy denoting whether subject has never been told that s/he has high blood pressure
Hunger	Dummy denoting whether subject indicated, when asked, whether s/he was hungry during the experiment
Time 9.30am	Dummy denoting whether subject attended a 9:30am experimental session
Time 11.30am	Dummy denoting whether subject attended an 11:30am experimental session
Time 2.30pm	Dummy denoting whether subject attended a 2.30pm experimental session
Mon	Dummy denoting whether subject's experimental session was on Monday
Tues	Dummy denoting whether subject's experimental session was on Tuesday

**Variable Name**	**Description of Variable**
Wed	Dummy denoting whether subject's experimental session was on Wednesday
Thurs	Dummy denoting whether subject's experimental session was on Thursday
